# A diet rich in fruit and whole grains is associated with a low risk of type 2 diabetes mellitus: findings from a case–control study in South China

**DOI:** 10.1017/S1368980020004930

**Published:** 2022-06

**Authors:** Yanbin Ye, Shuyu Zhuo, Wei Lu, Kaiyin He, Yi Sui, Yanbing Li, Yuming Chen, Shangling Wu, Peiyan Chen, Shi Fang

**Affiliations:** 1 Department of Clinical Nutrition, The First Affiliated Hospital of Sun Yat-sen University, 58 # Zhongshan Road 2, Guangzhou 510080, Guangzhou, China; 2 Department of Gastroenterology, The First Affiliated Hospital of Jinan University, Guangzhou, Guangzhou, China; 3 Department of Endocrinology, The First Affiliated Hospital of Sun Yat-sen University, Guangzhou, Guangzhou, China; 4 Department of Medical Statistics and Epidemiology, School of Public Health, Sun Yat-sen University, 74# Zhongshan Road 2, Guangzhou 510089, Guangzhou, China

**Keywords:** Type 2 diabetes mellitus, Dietary pattern, Fruits, Whole grains, China

## Abstract

**Objective:**

Various foods are associated with or protect against type 2 diabetes mellitus (T2DM). This study was to examine the associations of foods and food patterns with the risk of T2DM in South China.

**Design:**

Case–control study.

**Setting:**

The dietary patterns were identified by a principal components factor analysis. Univariable and multivariable conditional logistic regression analyses were used to analyse the associations between food groups and dietary patterns and the risk of T2DM.

**Participants:**

A total of 384 patients with T2DM and 768 controls.

**Results:**

After adjustment for total energy intake, the standard intake of grains (228·3 ± 71·9 *v*. 238·8 ± 73·1 g/d, *P* = 0·025) and fruits (109 ± 90 *v*. 145 ± 108 g/d, *P* < 0·001) were lower in T2DM than in controls. Four dietary patterns were identified: (1) high light-coloured vegetables and low grains, (2) high fruits, (3) high red meat and low grains and (4) high dark-coloured vegetable. After adjustment for covariables, multivariable conditional logistic regression analyses showed significant dose-dependent inverse associations between total fruit intake, whole grains intake and the score of the high-fruit dietary pattern (all *P*_for trend_ < 0·001) and the risk of T2DM. The adjusted OR (95 % CI) for T2DM comparing the extreme quartiles were 0·46 (0·29, 0·76) for total fruits, 0·48(0·31, 0·77) for whole grains and 0·42 (0·26, 0·68) for the high-fruit dietary pattern, respectively. Similar associations were observed for all subgroups of fruits (dark-colour and light-colour).

**Conclusion:**

In South China, a diet rich in fruit and whole grains is associated with lower risk of T2DM.

Type 2 diabetes mellitus (T2DM) is a common endocrine disorder characterised by variable degrees of insulin resistance and deficiency, resulting in hyperglycaemia^(1)^. Potential complications of T2DM include CVD, neuropathy, nephropathy, retinopathy and mortality^(1)^. The worldwide prevalence of T2DM was 9 % in men and 7·9 % in women in 2014^(1)^. In China, the prevalence of T2DM increased from 2·5 % in 1994^(2)^ to 10·9 % in 2013, with a prevalence of pre-diabetes of 35·7 %^(3)^. Medical expenses related to T2DM also exploded in China, from USD 0·25 billion in 1993 to USD 8·65 billion in 2009^(4)^.

The risk factors for T2DM include obesity, metabolic syndrome, poor diet and lack of exercise^(5)^. Especially, the westernisation of the diet in China over recent decades has been associated with the prevalence of T2DM^(6)^. A number of specific foods have been associated with T2DM, including processed meat^(7)^, red meat^(8)^, fried food^(9)^, white rice^(10)^ and sugar-sweetened beverages^(11)^, while higher consumption of cereal fibres^(12)^, fish^(13)^, milk^(14)^ and soyabeans (and their isoflavones)^(15)^ are associated with the prevention of T2DM and its complications. Regarding fruits and vegetables, their association with T2DM might be only significant for the lowest quintile^(16)^.

Nevertheless, the studies about the association between dietary patterns and T2DM often report variable and even conflicting results. Indeed, various tools for dietary assessment are available, each with their pros, cons and biases, and they yield variable results^(17)^. Dietary habits are highly cultural, and those cultural dietary patterns are complex and specific^(18)^. T2DM is a complex trait, influenced both by the environment and genetics^(19)^. Since the genetic patterns vary widely across the world, different populations will have a different genetic susceptibility to T2DM^(20)^. Therefore, the results of the available studies cannot be generalised to other populations, and studies specific to each population must be conducted in order to improve the understanding of the dietary risk of T2DM in specific populations. A meta-analysis of 16 cohorts showed that adhering to the Mediterranean diet, Dietary Approach to Stop Hypertension (DASH), or Alternative Healthy Eating Index (AHEI) was associated with a lower risk of T2DM, while dietary patterns characterised by meat (red and processed), refined grains, high-fat dairy products, eggs and fried foods were associated with a higher risk of T2DM^(21)^.

Therefore, the aim of the present study is to determine whether particular foods and dietary patterns have associations with T2DM risk in South China. The results could help guide the residents’ daily diet to prevent T2DM.

## Methods

### Study design and subjects

This was a case–control study of permanent adult residents (30–80 years of age) who had been living in Guangzhou (South China) for at least 5 years. This study was conducted according to the guidelines laid down in the Declaration of Helsinki and all procedures involving human subjects were approved by the ICE for Clinical Research and Animal Trials of the First Affiliated Hospital of Sun Yat-sen University. Written informed consent was obtained from all subjects.

From May 2011 to February 2012, most patients with new-onset T2DM at the endocrinology clinic and inpatient ward of the First and Second Affiliated Hospitals of Sun Yat-sen University were recruited. T2DM was confirmed based on their glucose examination reports. Control subjects were recruited using a questionnaire survey and fasting blood glucose (FBG) screening at Nonglin Street Community, Yuexiu District, Guangzhou, and from the otolaryngology department of the First and Second Affiliated Hospitals of Sun Yat-sen University.

The patients with T2DM met the diagnostic criteria of T2DM from the American Diabetes Association in 2007^(22)^ and were with new-onset T2DM, that is, ≤3 months since diagnosis. The control group included adults without abnormal glucose metabolism (FBG < 5·6 mmol/l and 2-h postprandial blood glucose < 7·8 mmol/l). The case–control ratio was 1:2, and the controls were matched with the T2DM group for sex and age (within ± 3 years).

The exclusion criteria were (1) severe heart, lung, liver or renal dysfunction, or severe gastrointestinal diseases; (2) thyroid or other endocrine dysfunction; (3) taking hormones; (4) paralysis or walking difficulties; (5) mental disorders or (6) vegetarians. Individuals with FBG of 5·6–7·0 mmol/l were excluded.

### Data collection and dietary assessment

A structured questionnaire that included sociodemographic characteristics, lifestyle habits, history of chronic diseases, medical history and dietary habits was completed by all subjects during a face-to-face interview by trained interviewers.

A validated dietary FFQ was used for dietary intake assessment^(23)^. It has seven categories of food, including 79 food items. The participants were asked to report the information on the frequency of each food item intake and its portion size in the last year before diagnosis (patients) or interview (control subjects). Food photographs of portion size were used to help estimate the amount of food consumption. According to the frequency of intake and portion size, the average consumption amount of each food item (g/d) was calculated. For seasonal foods, participants were asked to report on how many months of the year they consumed each item. The average daily intake of each food group and its nutrients was calculated according to the China Food Composition 2002^(24)^. The participants were excluded if their FFQ was invalid, that is, more than five food items were missing or if the FFQ resulted in implausible energy intakes (< 800 or > 4000 kcal per day). To reduce the complexity of the food data, the 79 food items were aggregated into 30 groups according to similarities in nutrients or processing methods to identify the dietary patterns (see online supplementary material, Supplemental Table S4). We used a questionnaire to estimate the usual physical activity, which including hours and minutes spent on daily activities and leisure activities in the recent 1 year. Total energy expenditure was evaluated according to metabolic equivalents ^(25)^.

Physical examinations (height, weight, waist and hip circumferences and blood pressure) and laboratory examinations (FBG for the controls) were examined on the interview morning.

### Statistical analysis

All data of the FFQ were managed using Epidata 3.0 software and exported to SPSS 16.0 (IBM) for statistical analyses. Two-sided *t* tests were used, and *P*-values < 0·05 were considered significant. To achieve an approximately normal distribution for statistical analysis, logarithmic transformation was applied to daily energy intake, whereas the square root transformation was applied to other dietary data.

To evaluate the sociodemographic characteristics, other potential risk factors, and food groups and nutrients, we used independent-samples *t* test (continuous variables) and chi-square test (categorical variables) for comparison between the case and control groups. All nutrients and food groups were adjusted by the residual energy method^(26)^. The principal component analysis was used for the construction of dietary patterns. The standardised intake values of the 30 food groups (see online supplementary material, Supplemental Tables S4 and S5) were included in the factor analysis. Four factors were retained based on an inspection of the plots. Varimax-rotated by orthogonal transformation was used to obtain the factor loading value of each factor. We calculated the factor scores for each retained factor by the summation of the intakes of each food group and weight by their factor loadings (a factor loading ≥ 0·1 showed the main component of the factor). For the analysis of the associations between dietary factors and T2DM, the intake of each food, nutrient and score of each dietary pattern was divided into quartiles (Q1–Q4, Q1 as the reference group) based on the distribution of dietary intake among the control group and then applied the gender-specific cut-offs to the case. The Cox conditional logistic regression was conducted with the bottom quartile group (Q1) as the reference group. Univariable and multivariable analyses were used to calculate the OR with the corresponding 95 % CI. The univariable analyses were not adjusted, while in multivariable analyses, 13 confounding factors were adjusted for (age, sex, marital status, educational level, occupation, smoking, alcohol consumption, tea drinking, economic conditions energy intake, physical activity, family history of T2DM and fibre intake (for dietary pattern only)). The stepwise method was used for including the covariables. The ordinal values for dietary intake and pattern categories were also included as continuous variables in the linear trend tests. The associations between the subcategories of grains, fruits and vegetables and the risk of T2DM were also evaluated in this study.

## Results

### Recruitment

**Fig. 1:**
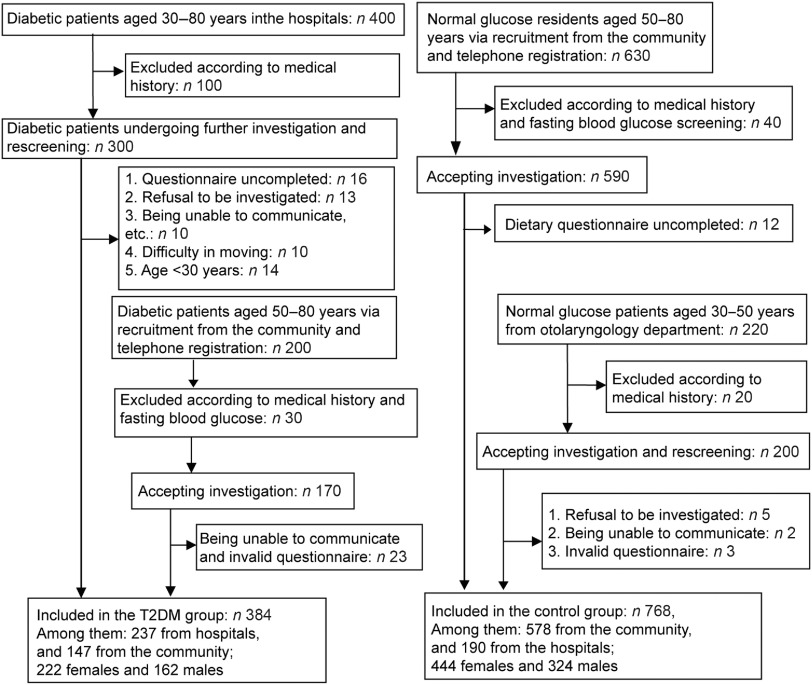
Patient flowchart. T2DM, type 2 diabetes mellitus

In this study, 600 diabetic patients (30–80 years of age) were recruited at the First and Second Affiliated Hospitals of Sun Yat-sen University and Mapenggang Community, Nonglin Street, Yuexiu District. After initial screening and rescreening, 384 eligible patients were enrolled. For the control group, 630 people from the community and 220 patients in other departments of the hospitals were recruited, matched for age and sex with those of the T2DM group. According to the inclusion/exclusion criteria, 768 subjects were included in the control group (578 from the community and 190 from the hospitals). The recruitment process and details are shown in Fig. 1.

### Characteristics of the subjects

Table 1 shows the sociodemographic characteristics of the subjects. Over 2/3 (66·6 % of the T2DM group and 68·0 % of the control group) of the subjects were 50–70 years of age. The frequency of T2DM was higher among subjects < 50 years of age. Compared with the control subjects, the subjects with T2DM were more likely to have a higher education level, household income, energy intake, BMI, alcohol consumption and the likelihood of having a family history of T2DM (34·4 % *v*. 11·2 %). The other characteristics of the subjects were not significantly different between the two groups.

**Table 1:** Demographic, lifestyle characteristics and selected T2DM risk factors of the study population in Guangzhou, China

	Control group		T2DM group		
Variables	*n*	%	*n*	%	*P* *
Sex					1·000
Female	444	57·8	222	57·8	
Male	324	42·2	162	42·2	
Age					1·000
30–40	102	13·3	50	13·1	
41–50	117	15·2	60	15·6	
51–60	307	40·0	153	39·8	
61–70	215	28·0	103	26·8	
71–80	27	3·5	18	4·7	
Educational level†					< 0·001
Primary education degree or below	95	12·4	81	21·1	
Junior high school	216	28·1	87	22·6	
Senior high school and technical secondary school	282	36·7	99	25·8	
College degree or above	175	22·8	117	30·5	
Household income, yuan/month /person†					< 0·001
≤ 500 yuan	37	4·8	13	3·4	
500–1500 yuan	254	33·1	67	17·4	
1501–3000 yuan	328	42·7	144	36·5	
≥ 3001 yuan	149	19·4	160	41·7	
Long-term occupation					0·051
Light worker	470	61·2	242	63·0	
Moderate worker	274	335·7	120	31·3	
Heavy worker	24	3·1	22	5·7	
Work status in the past year					0·484
Full-time	247	32·2	137	35·7	
Part-time	29	3·8	13	3·4	
Retired/non-permanent	492	64·0	234	60·9	
Marital status					0·102
Married or cohabiting	680	88·5	252	92·1	
Widowed, unmarried and divorced	88	11·5	32	7·9	
Family history ofT2DM					< 0·001
Yes	86	11·2	132	34·4	
No	682	88·8	252	65·6	
Smoking‡					0·103
Yes	182	23·7	108	28·1	
No	586	76·3	276	71·9	
Passive smoking§					0·294
Yes	235	30·6	106	27·6	
No	533	69·4	278	72·4	
Alcohol consumption||					0·014
Yes	73	9·5	55	14·3	
No	695	90·5	329	82·7	
Tea drinking¶					0·196
Yes	391	50·9	211	55·0	
No	377	49·1	173	45·0	
Source of subjects					< 0·001
Hospital	190	24·7	237	61·7	
Community	578	75·3	147	38·3	
Dietary, activity and physical characteristics	Mean				
Energy intake (kJ/d)					< 0·001
Mean	9075		7965		
sd	2675		2345		
Physical activity(MET-h/d)					0·359
Mean	34·6		34·1		
sd	9·1		7·6		
BMI (kg/m^2^)					< 0·001
Mean	22·9		24·2		
sd	3·1		3·6		

T2DM, type 2 diabetes mellitus; MET-h/d, metabolic equivalent hours per day.

* The difference in the distribution of categorical variables between the two groups was tested by the chi-square test; the difference in continuous variables was tested using the *t* test.

† The two independent-samples rank-sum test was used for comparison of the difference in the distribution of education level and family monthly income per capita between the two groups.

‡ Smokers referred to those who smoked at least one cigarette per day for more than half a year.

§ Passive smokers referred to those living in smoking environments with an average of more than one cigarette or 5 min/d.

|| Drinkers referred to those who drank at least once a week for six consecutive months.

¶ Tea drinkers referred to those who had tea at least twice a week.

### Daily average intake of food categories and nutrients

Compared with controls, patients with T2DM had a lower intake of total grains (228·3 ± 71·9 *v*. 238·8 ± 73·1 g/d, *P* = 0·025) and fruits (109 ± 90 *v*. 145 ± 108 g/d, *P* < 0·001), a higher intake of animal foods and cooking oil (243 ± 129 *v*. 229 ± 95 g/d, *P* = 0·047; and 26·1 ± 12·0 *v*. 17·8 ± 11·6 g/d, *P* < 0·001, respectively), and a similar intake of milk, soybean and derivatives intake, and other food groups (Table 2).

**Table 2:** Daily average intake of main food categories and nutrients of the study population in Guangzhou, china

	T2DM group (*n* 384)		Control group (*n* 768)		
Daily average intake	Mean	sd	Mean	sd	*P* *
Main food categories					
Total intake of grains, g	228·3	71·9	238·1	75·1	0·025
Total intake of soyabeans and related products, g	4·7	6·3	4·7	10·4	0·905
Total intake of vegetables, g	386	282	356	165	0·024
Total intake of fruits, g	109	90	145	108	< 0·001
Total intake of animal foods, g	243	129	229	95	0·047
Total intake of eggs, g	26·9	19·6	26·4	18·6	0·663
Total intake of milk, g	126	138	127	142	0·924
Total intake of fungi and seaweeds, g	11·8	34·9	9·5	11·3	0·095
Total intake of nuts, g	13·8	26·7	16·8	26·7	0·082
Total intake of cooking oil, g	26·1	12	17·8	11·6	< 0·001
Nutrients					
Energy, kJ	9075	2675	7965	2345	< 0·001
Carbohydrate, g	284·7	90·9	277·3	85·4	0·261
Total protein, g	92·5	33·1	82·7	28·2	< 0·001
Animal protein, g	51·9	28	44·2	22·1	< 0·001
Vegetable protein, g	38·5	11·9	37·9	7·8	0·273
Soyabean protein, g	2·2	2·5	2·4	2·9	0·384
Fat, g	78·6	19·9	57·2	16·7	< 0·001
Dietary fibre, g	12·2	3·5	11·4	3·3	< 0·001
Total isoflavone, mg	10·3	10·6	10·6	10·7	0·316
Daidzein, mg	4·3	4·4	4·4	4·5	0·408
Genistein, mg	5·8	6·1	6·9	6·0	0·202
Glycitein, mg	0·56	0·67	0·6	0·8	0·812
VitC, mg	176·2	81	182·6	70·8	0·074
Carotene	5814	3030	5625	2720	0·041
Mg, mg	341·2	114·2	339·3	94·2	0·001
Zn, mg	17·6	7·5	17·3	5·4	< 0·001
Se, mg	64·7	36·9	65·2	31·9	0·007

T2DM, type 2 diabetes mellitus.

* Derived from *t* test.

† Mean daily intake.

‡ Standard deviation.

The average energy intake for the T2DM and control groups was 9075 ± 2675 kJ/d and 7965 ± 2345 kJ/d, respectively (*P* < 0·001). The average intake of total protein and fat in the T2DM group was higher than those of the control group (92·5 ± 33·1 *v*. 82·7 ± 28·2 g/d, 78·6 ± 19·9 *v*. 57·2 ± 16·7 g/d, both *P* < 0·001), and the intake of animal protein was higher in the diabetic patients (51·9 ± 28·0 *v*. 44·2 ± 22·1, *P* < 0·001) (Table 2).

### Association between six main food categories and risk of type 2 diabetes mellitus

Table 3 shows the associations between the six food categories and the risk of T2DM. The intake of total grains in women was lower than that of men, while the milk intake was lower in men than in women. Univariable and multivariable conditional logistic regression analyses showed significant dose-dependent inverse correlations between the intake of fruits and milk and the risk of T2DM in all subjects, without and with adjustment for confounders (*P*_for trend_ < 0·05–0·01). After adjustment for age, sex and 11 other potential factors, the significant associations still remained strong in model 2: the OR (95 % CI) of T2DM for the highest quartile, compared with the lowest quartile, were 0·46 (0·29, 0·72) for fruits and 0·51 (0·33, 0·81) for milk. An inverse association was also seen between an appropriate range of intake of total grains and the risk of T2DM, and the OR (95 % CI) for the second and the third quartile were 0·64 (0·41, 0·99) and 0·55 (0·35, 0·84) (*P*_for trend_ = 0·081). The intake of cooking oil significantly increased the risk of T2DM with a strong dose-dependent effect (P_for trend_ < 0·001) in both regression models, and the OR (95 % CI) of T2DM after adjustment for all confounding factors for the highest quartile was 10·85 (6·14, 19·17). The intake of vegetables and animal foods did not show any effect on the risk of T2DM in the study population.

**Table 3:** OR of T2DM for quartiles of six main food groups intake in Guangzhou, China

	Quartiles of dietary energy-adjusted intake							
		Q2		Q3		Q4 (highest)		
Food group	Q1 (referent)	OR	95 % CI	OR	95 % CI	OR	95 % CI	*P*_for trend_
Total grains								
*Intake (male/female), g/d	202/148	266/186		312/214		354/264		
*n* (case–control)	141/192	69/192		67/192		107/192		
ORI†	1·00	0·50	0·35, 0·71	0·50	0·35, 0·71	0·78	0·56, 1·08	0·062
ORII‡	1·00	0·64	0·41, 0·99	0·55	0·35, 0·84	0·73	0·48, 1·12	0·081
Fruits								
*Intake (male/female), g/d	33/38	87/99		146/156		256/268		
*n* (case–control)	165/192	83/192		66/192		70/192		
ORI†	1·00	0·49	0·36, 0·68	0·40	0·28, 0·57	0·41	0·28, 0·58	< 0·001
ORII‡	1·00	0·49	0·32, 0·75	0·41	0·26, 0·63	0·46	0·29, 0·72	< 0·001
Vegetables								
*Intake (male/female), g/d	196/188	291/285		388/385		538/537		
*n* (case–control)	88/192	95/192		99/192		102/192		
ORI†	1·00	1·07	0·76, 1·52	1·12	0·80, 1·58	1·16	0·82, 1·64	0·389
ORII‡	1·00	1·44	0·92, 2·26	1·27	0·83, 1·94	1·38	0·90, 2·12	0·222
Animal foods								
*Intake (male/female), g/d	155/122	227/173		284/219		386/301		
*n* (case–control)	109/192	79/192		81/192		115/192		
ORI†	1·00	0·73	0·51, 1·03	0·76	0·54, 1·07	1·06	0·76, 1·46	0·715
ORII‡	1·00	0·87	0·57, 1·33	1·00	0·66, 1·51	1·08	0·72, 1·63	0·632
Milk								
*Intake (male/female), g/d	2·2/4·3	21·8/71·8		83·9/172·6		266·8/298·0		
*n* (case–control)	125/192	67/192		95/192		97/192		
ORI†	1·00	0·52	0·36, 0·75	0·76	0·54, 1·05	0·71	0·53, 1·07	0·292
ORII‡	1·00	0·52	0·33, 0·84	0·64	0·41, 0·99	0·51	0·33, 0·81	0·012
Cooking oil								
*Intake (male/female), g/d	8·0/8·3	13·7/11·6		21·5/17·7		31·9/28·2		
*n* (case–control)	29/192	38/192		94/192		223/192		
ORI†	1·00	1·20	0·69, 2·08	3·11	1·93, 5·00	7·82	4·97, 12·31	< 0·001
ORII‡	1·00	1·38	0·70, 2·74	4·04	2·25, 7·24	10·85	6·14, 19·17	< 0·001

T2DM, type 2 diabetes mellitus.

* Median intake in male/female controls at each quantile after energy-adjusted.

† No factor adjusted.

‡ Adjusted for age, sex, marital status, educational level, occupation, smoking, alcohol consumption, tea drinking, economic conditions, energy intake, physical activity and family history of T2DM.

### Association between grain subcategories and risk of type 2 diabetes mellitus

The associations between the subgroups of grains and the risk of T2DM were also tested (see online supplementary material, Supplemental Table S1). Total grains were divided into three subgroups: whole grains (with whole-wheat bread as the representative member), high-energy grains(Chinese cakes such as radish cakes and water chestnut cakes, cakes, fried snacks and biscuits) and refined grains (rice (including glutinous rice products), porridge, oatmeal, corn flakes, noodles, white bread or steamed bun, white bread or hamburger, barbecued pork buns, and dumplings). The results showed that both univariable and multivariable conditional logistic regression analyses showed a significant dose-dependent inverse correlation between the intake of whole grains and the risk of T2DM (*P*_for trend_ ≤ 0·001), and the reduction of the risk after adjustment for the confounding factors for the highest quartile *v*. the lowest quartile was 52 % (95 % CI: 23 %, 69 %). There was no association found between high-energy grains and refined grains and the risk of T2DM.

### Association between vegetable and fruit subcategories and risk of type 2 diabetes mellitus

Table 4 and Supplementary Table 2 show the analyses of the associations between vegetable and beans and the risk of T2DM, with the results of subgroup analyses for different kinds of fruit shown in the Supplementary Table S3. Both two logistic regression analyses showed significant dose-dependent inverse associations between the intake of light- and dark-coloured fruit subgroups with the risk of T2DM (*P*_for trend_ < 0·05), and this inverse association was similar to that observed for total fruits (Tables 2 and 3). After adjustment for the potential confounding factors, the OR (95 % CI) of T2DM for the highest quartile, compared with the lowest quartile, was 0·58 (0·37, 0·90) for dark-coloured fruit and 0·39 (025, 0·60) for light-coloured fruit. The results of analyses of the light- and dark-coloured vegetable subgroups were the same as that for the total vegetables (Table 2 and 3), and their intakes were not correlated with the risk of T2DM. Univariable analyses showed a significant inverse dose-dependent effect of the intake of root vegetables with the risk of T2DM (*P*_for trend_ < 0·01), but this protective effect disappeared after adjusting for the confounding factors. The significant dose-dependent inverse correlations between the intake of fresh beans and mixed beans and the risk of T2DM were found in univariable conditional logistic regression analyses or multiple adjustment models (models 2, *P*_for trend_ < 0·01). The results in Supplementary Table S3 indicated null significant difference between different categories of fruits with the risk of T2DM (all *P*_for trend_ > 0·05).

**Table 4:** OR of T2DM for quartiles by subgroups of vegetables and fruits in Guangzhou, China

	Quartiles of dietary energy-adjusted intake							
		Q2		Q3		Q4 (highest)		
Food group	Q1 (referent)	OR	95 % CI	OR	95 % CI	OR	95 % CI	*P*_for trend_
Dark-coloured vegetables								
Intake* (male/female), g/d	138/124	208/199		285/275		419/400		
*n* (case–control)	93/192	91/192		100/192		100/192		
ORI†	1·00	0·98	0·69, 1·38	1·07	0·76, 1·51	1·08	0·76, 1·53	0·561
ORII‡	1·00	0·89	0·57, 1·38	1·18	0·77, 1·83	1·47	0·97, 2·23	0·084
Light-coloured vegetables								
Intake* (male/female), g/d	44/48	101/97		165/159		289/279		
*n* (case–control)	108/192	111/192		79/192		86/192		
ORI†	1·00	1·03	0·74, 1·43	0·73	0·52, 1·04	0·80	0·56, 1·13	0·069
ORII‡	1·00	1·47	0·95, 2·27	1·29	0·83, 2·00	1·33	0·84, 2·11	0·364
Root vegetables								
Intake* (male/female), g/d	10/9	27/26		50/46		101/95		
*n* (case–control)	159/192	84/192		76/192		65/192		
ORI†	1·00	0·72	0·52, 1·00	0·66	0·48, 0·91	0·66	0·46, 0·93	0·007
ORII‡	1·00	0·96	0·65, 1·44	0·80	0·54, 1·19	0·76	0·49, 1·18	0·099
Dark-coloured fruits								
Intake* (male/female), g/d	7/9	21/25		42/46		105/100		
*n* (case–control)	136/192	93/192		80/192		75/192		
ORI†	1·00	0·68	0·48, 0·96	0·58	0·42, 0·83	0·54	0·38, 0·77	< 0·001
ORII‡	1·00	0·71	0·47, 1·08	0·50	0·33, 0·77	0·58	0·37, 0·90	0·003
Light-coloured fruits								
Intake* (male/female), g/d	20/19	54/53		96/94		175/178		
*n* (case–control)	137/192	91/192		76/192		80/192		
ORI†	1·00	0·52	0·31, 0·85	0·39	0·24, 0·64	0·37	0·22, 0·61	< 0·001
ORII‡	1·00	0·45	0·29, 0·70	0·40	0·26, 0·63	0·39	0·25, 0·60	0·003

T2DM, type 2 diabetes mellitus.

* Median intake in male/female controls at each quantile after energy-adjusted.

† No factor adjusted.

‡ Adjusted for age, sex, marital status, educational level, occupation, smoking, alcohol consumption, tea drinking, economic conditions, energy intake, physical activity and family history of T2DM.

### Association between dietary patterns score and risk of type 2 diabetes mellitus

We used the principal component analysis to retain the four main factors associated with T2DM. The details of food composition for 30 food groups and the factor loadings associated with each pattern are given in Supplementary Tables S4 and S5. Each pattern was labelled according to the food groups with high absolute loadings scores. These four factors accounted for 36·94, 13·08, 10·37 and 7·83 % of the variability, respectively, and 68·22 % of the variance in food intake overall. The first factor was a high intake of light-coloured vegetables, root vegetables, nuts, whole milk, and eggs and a low intake of grains, which was named the high-light-coloured vegetables and low-grain dietary pattern. The second factor was characterised by a high intake of fruits and freshwater fish and a low intake of cooking oil and was named the high-fruit dietary pattern. The third factor was characterised by a high intake of red meat and a low intake of grains and was named the high-meat and low-grain dietary pattern. The fourth factor was characterised by a high intake of dark-coloured vegetables and was named the high-dark-coloured vegetable dietary pattern.

Univariable conditional logistic regression analyses without correction for any factor showed a dose-dependent inverse association between the risk of T2DM and the high-fruit pattern (*P*_for trend_ < 0·001), but a marginally significant positive association with the high-red meat and low-grain dietary pattern (*P*_for trend_ = 0·053, Q4 *v*. Q1: OR = 1·46, 95 % CI 1·02, 2·09) (Table 5). After adjustment for age, sex, marital status, educational level, occupation, smoking, alcohol consumption, tea drinking, economic conditions, energy intake, physical activity, family history of T2DM and dietary fibre, the significant association remained between the high-fruit dietary pattern and the risk of T2DM (model 2 and 3, *P*_for trend_ < 0·001), but not for the high-red meat and low-grain dietary pattern (*P*_for trend_ = 0·072). The significant inverse correlation between the high-fruit dietary pattern and the risk of T2DM was observed, and, compared with the lowest quartile, the risk of T2DM for the highest quartile was reduced (OR = 0·40, 95 % CI: 0·25, 0·63, *P*_for trend_ < 0·001). There was no significant association between the risk of T2DM and the high-light-coloured vegetable and low-grain dietary pattern and the high-dark-coloured vegetable dietary pattern.

**Table 5:** OR of T2DM for quartiles of dietary patterns in Guangzhou, China

	Quartiles of dietary energy-adjusted intake							
		Q2		Q3		Q4 (Highest)		
Dietary pattern	Q1 (Referent)	OR	95 % CI	OR	95 % CI	OR	95 % CI	*P*_for trend_
High-light-coloured vegetable and low-grain								
*n* (case–control)	93/192	94/192		97/192		100/192		
ORI*	1·00	0·96	0·66, 1·40	1·07	0·73, 1·56	1·02	0·70, 1·47	0·801
ORII†	1·00	1·09	0·69, 1·73	1·43	0·90, 2·27	1·22	0·76, 1·96	0·259
ORIII‡	1·00	1·24	0·77, 1·99	1·67	1·03, 2·69	1·59	0·96, 2·63	0·860
High-fruit								
*n* (case–control)	143/192	149/192		88/192		67/192		
ORI*	1·00	0·53	0·37, 0·76	0·48	0·33, 0·69	0·39	0·27, 0·57	<0·001
ORII†	1·00	0·49	0·32, 0·77	0·50	0·32, 0·79	0·42	0·26, 0·68	<0·001
ORIII‡	1·00	0·46	0·30, 0·71	0·48	0·31, 0·74	0·40	0·25, 0·63	<0·001
High-red meat and low-grain								
*n* (case–control)	91/192	87/192		78/192		128/192		
ORI*	1·00	0·96	0·66, 1·39	0·87	0·59, 1·27	1·46	1·02, 2·09	0·053
ORII†	1·00	0·88	0·56, 1·39	0·93	0·59, 1·46	1·53	1·00, 2·35	0·056
ORIII‡	1·00	0·89	0·56, 1·40	0·93	0·59,1·47	1·50	0·97, 2·32	0·072
High-dark-coloured vegetable								
*n* (case–control)	91/192	78/192		96/192		119/192		
ORI*	1·00	0·82	0·55, 1·21	0·97	0·68, 1·40	1·32	0·92, 1·89	0·078
ORII†	1·00	0·89	0·55, 1·43	1·00	0·64, 1·56	1·52	0·98, 2·35	0·075
ORIII‡	1·00	1·03	0·63, 1·69	1·33	0·82, 2·13	2·72	1·63, 4·54	0·074

T2DM, type 2 diabetes mellitus.

* No factor adjusted.

† Adjusted for age, sex, marital status, educational level, occupation, smoking, alcohol consumption, tea drinking, economic conditions, energy intake, physical activity and family history of T2DM.

‡ Adjusted for the covariates in model 2 plus dietary fibre.

## Discussion

Various foods are associated with or protect against T2DM, but the dietary patterns are often population-specific. This study aimed to assess the associations between the foods and dietary patterns and the risk of T2DM in South China. The results suggest that in subjects from South China, a high-fruit dietary pattern is associated with a lower risk of T2DM.

In this study, conditional logistic regression analyses were performed to investigate the associations between the intake of different food categories and the risk of T2DM. In addition, the factor analysis method was used to construct possible dietary patterns, and the scores were used as the dietary evaluation method to explore the correlation between the overall quality of diet and the risk of T2DM. The results showed that a dietary pattern characterised by a moderate intake of grains and carbohydrates, high intake of whole grains, various fruits, milk, fresh beans and mixed dry beans could reduce the risk of T2DM. Among them, whole grains, fruits and milk had more significant protective effects, and the higher the intake, the stronger the protective effect. Refined grains with more cooking oils and carbohydrates, high meat intake, and low grain intake may increase the likelihood of developing T2DM, which needs further confirmation. In fact, the protective dietary pattern described above (whole grains, various fruits, milk, fresh beans, mixed beans and high fruit) is similar to the Mediterranean dietary pattern (olive oil, cereals, fruits, vegetables, nuts, seeds, fish, seafood and dairy)^(27)^, which is already well known to be associated with a decreased risk of T2DM^(28)^. On the other hand, the dietary pattern observed here as being associated with an increased risk of T2DM (refined grains with more cooking oils and carbohydrates, high meat intake, and low grain intake) is similar to the Western dietary pattern (fried foods, refined grains, and processed and red meat)^(29)^, which is already well known to be associated with the epidemics of obesity, diabetes and metabolic syndrome in Western countries^(30)^. Supporting these results, a recent Chinese study showed that dietary patterns rich in refined grains increased the risk of T2DM, while those rich in whole grains decreased the risk of T2DM^(31)^. A study from Zhejiang Province (East China) showed that the Western diet was associated with an increased risk of T2DM, while a dietary pattern characterised by grains and vegetables was associated with a lower risk^(32)^. The Singapore Health Study revealed that a dietary pattern characterised by vegetables, fruits and soya foods was beneficial against T2DM, while a high intake of dim sum, meat, sweetened food/beverages and fried foods was associated with a high risk of T2DM^(33)^. Those results are supported by two meta-analyses that showed that whole grains, fruits and milk products decreased the risk of T2DM, while red meat, processed meat and sugar-sweetened beverages increased this risk^(34,35)^. A combined analysis of the Nurses’ Health Study, Nurses’ Health Study 2 and Health Professionals’ Follow-Up Study also supports those findings, that is, that high intake of coffee, whole grains, fruits and nuts is associated with a decreased risk of T2DM, while a high intake of meats, refined grains and sweetened beverages increased the risk^(36)^. Similar, but not identical, dietary patterns have been observed by other studies^(5,21,35)^. Nevertheless, caution must be taken when comparing dietary patterns from all around the globe because of the cultural differences in diet that cannot be taken into account by the food questionnaires.

There is increasing evidence that the intake of whole grains in substitution to refined grains reduces the risk of T2DM^(37)^. Whole grains have a high content of dietary fibres that contribute to decreasing the energy density and higher gastric distention and to increase hormones associated with energy and glucose homoeostasis^(38)^. The structural integrity of whole grains requires a higher chewing rate, leading to earlier satiety that benefits body weight control^(39)^. A meta-regression analysis showed that consuming 45 g/d of whole grains would reduce the T2DM risk by 20 % compared with 7·5 g/d^(40)^. In the present study, patients with T2DM consumed less whole-grain foods compared with controls, but the amount was relatively low in both groups.

The results of previous studies regarding vegetable and fruit intakes and the risk of T2DM are inconsistent, but the majority reported protective effects. Although Harding *et al.*^(16)^ showed that the association between fruit and vegetable intake with T2DM was only significant for the lowest quintile, a meta-analysis by Li *et al.*^(41)^ showed that a high intake of fruits or green leafy vegetables was associated with a markedly lower risk of T2DM. Shu *et al.*^(32)^ showed that vegetables, not fruits, were associated with a decreased risk of T2DM, while Odegaard *et al.*^(33)^ showed that both vegetables and fruits were protective against T2DM. Many dark green vegetables and fruits (except bananas) have high levels of carotenoids but contain little or no starch, whereas avocado, maize and potatoes are full of starch, indicating the importance of dividing the vegetables or fruits into separate categories to avoid the influence of different nutrient content^(38)^. Consistent with the meta-analysis study by Wang PY *et al.*^(42)^, both of us have found null association between intake of total vegetables with T2DM, but there were inconsistent and insignificant associations between green leafy, yellow, cruciferous vegetables and diabetes in our study, and the different findings of total vegetables and specific vegetables strengthened the potential heterogeneity due to the difference in the vegetables categories of subgroup analyses between their study and ours. On the one hand, vegetables and fruits have been placed in dietary guidance due to their high concentrations of vitamins, antioxidants, minerals, phytochemicals and dietary fibres^(38)^. On the other hand, vegetables and fruits are good sources of K, and their energy density is low; the mechanism in the prevention of T2DM could be similar to that of whole grains and beans^(41)^, but studies of fruit fibres on T2DM yielded inconsistent results^(43)^. Fruits are also rich in micronutrients, especially vitamins and antioxidants^(44)^, which are well known to protect against the development of T2DM. The Mg contained in fruits is also inversely correlated with the risk of diabetes^(45)^. Fruits have a low energy density, contributing to weight loss^(16)^. In the present study, fruit intake is the strongest contributor to Pattern 2 against T2DM. This is supported by a Chinese longitudinal study that showed that fresh fruit intake protects against incident diabetes^(46)^.

The present study showed that beans and dairy were part of the dietary pattern for protection against T2DM, while meat (especially red meat intake) was associated with an increased risk of T2DM. Replacing white rice by beans has been shown to improve cardiometabolic indicators of T2DM^(47)^. Beans also contain fibres, which act in the same way as fibres from whole grains. Dairy contains a wide variety of vitamins and minerals that could play roles in the prevention of T2DM^(48)^, and bacteria contained in dairy products like yogurt and cheese also play roles in glucose homoeostasis^(49)^. The present study found total milk intake was associated with a low risk of T2DM, while Marangoni F *et al.*^(50)^ indicate that the protective association between milk and diabetes seems to apply to low-fat milk but not to full-fat milk. The inconsistent results might be due to the low consumption of total milk among Chinese populations, especially low intake of low-fat milk and skim milk^(51)^. Red meat and processed meat have been reported to have a positive correlation with T2DM in a number of studies, and processed meats are also known to be unhealthy^(21,34,35,52)^. Although the exact mechanisms through which meats increase the risk of T2DM is unknown, it is known that body weight can be affected by specific amino acids found in meat, as well as by SFA^(52)^.

A dietary pattern characterised by whole grains, various fruits, milk, fresh beans, mixed beans and high fruit has been suggested to be used in the management of T2DM. Indeed, a Chinese longitudinal study showed that fresh fruit intake protects against vascular complications of T2DM^(46)^. A high intake of fruits is also recommended by the dietary guidelines for T2DM^(53)^. Furthermore, the Mediterranean diet not only protects against T2DM but it is also suggested as part of the management of T2DM^(27)^. Actually, the Mediterranean diet has been shown to improve glycemic control in T2DM^(54)^. This shows the importance of consuming healthy foods, which should decrease the mortality and morbidity of T2DM, saving lives and financial resources.

This study has some strengths. This was a T2DM case–control study with strictly implemented inclusion and exclusion criteria. New-onset diabetic patients (those diagnosed within 3 months of enrolment) were included in the study, and those with recently measured normal values of FBG were included as controls. This measure improved the representativeness of cases and prevented changes in dietary habits due to T2DM. For the T2DM and control groups, patients with diseases or conditions that may affect outcomes (such as insulin resistance) were excluded, thus minimising the potential confounding factors. The FFQ that we used has shown good reliability and validity for dietary assessment, as validated in a local female population^(23)^. Finally, the combination of a single nutrient, food group and dietary pattern was used to explore and verify factors that may protect against T2DM in the diet, which was also beneficial to the direct promotion and application of this research findings.

This study also has some limitations. Given it was a case–control study, there may have selection bias and unclear causal relationships, but we tried to reduce this bias by recruiting the case patients and control subjects from a variety of sources (two hospitals and one community). A FFQ was used to investigate the dietary intake of new-onset diabetic patients and control subjects over the past year, and recall bias is inevitable^(55)^, but this recall error is unlikely to differ between the patients and control subjects. Moreover, there may be a selection bias in self-reported information by the patients with T2DM, since patients tend to change their dietary habits after diseases, but adults generally maintain a long-term stable dietary habit^(56)^, and we included only incident cases and meticulously excluded participants with a history of other chronic diseases that might change dietary habits or nutritional factors, and our dietary intake assessment covering the year prior to diagnosis or interview was judged to reflect the participants’ long-term dietary habits and was unlikely to produce an inverse causal relationship. Finally, the subjects of this study were all from the main geographical area (South China), which limits generalisability.

In conclusion, in Chinese subjects from Guangzhou, fruit intake has protective effects against T2DM. A high-fruit dietary pattern is associated with a lower risk of T2DM. Other dietary factors that protected against T2DM included whole grains, milk, fruits, total grains and an appropriate amount of vegetables. Large and well-designed studies are recommended to confirm this conclusion.

## Supplementary material

For supplementary material accompanying this paper visit https://doi.org/10.1017/S1368980020004930. click here to view supplementary material
