# Supplementary material for: A diet rich in fruit and whole grains is associated with a low risk of type 2 diabetes mellitus: findings from a case–control study in South China
Source: Public Health Nutr. 2020 Dec 15;25(6):1492–503. doi: 10.1017/S1368980020004930 (PMC9991751; doi:10.1017/S1368980020004930)
Supplement: Supplementary file 1 [file S1368980020004930sup.zip › S1368980020004930sup001.docx]

**Supplementary Table S1.** Odds ratios (95%CIs) of T2DM for quartiles by subgroups of grains in Guangzhou, China

|  | Quartiles of dietary energy-adjusted intake | | | |  |
| --- | --- | --- | --- | --- | --- |
| Food group | Q1(Referent) | Q2 | Q3 | Q4 (Highest) | *p* - trend |
| Total grains |  |  |  |  |  |
| Whole grains |  |  |  |  |  |
| ^a^Intake (M/F), g/d | 0.0/0.0 | 0.0/0.2 | 1.5/2.9 | 8.7/11.6 |  |
| N(case/control) | 157/192 | 78/192 | 82/192 | 67/192 |  |
| ^b^ ORⅠ(95%CI) | 1.00 | *0.46 (0.33,0.66)* | *0.48 (0.34,0.69)* | *0.39 (0.27,0.57)* | *<0.001* |
| ^c^ ORⅡ(95%CI) | 1.00 | *0.83 (0.51,1.35)* | *0.64 (0.40,1.01)* | *0.48 (0.31,0.77)* | *0.001* |
| High-energy grains |  |  |  |  |  |
| ^a^Intake(M/F), g/d | 0.2/1.2 | 3.6/4.8 | 10.1/10.5 | 26.7/25.2 |  |
| N(case/control) | 83/192 | 109/192 | 99/192 | 93/192 |  |
| ^b^ ORⅠ(95%CI) | 1.00 | *1.34 (0.93,1.93)* | 1.26 (0.88,1.81) | 1.01 (0.70,1.44) | 0.837 |
| ^c^ ORⅡ(95%CI) | 1.00 | 1.55 (0.99,2.42) | 1.24 (0.80,1.94) | 1.05 (0.68,1.63) | 0.889 |
| Refined grains |  |  |  |  |  |
| ^a^Intake (M/F), g/d | 163/122 | 237/168 | 302/204 | 368/283 |  |
| N(case/control) | 96/192 | 101/192 | 95/192 | 92/192 |  |
| ^b^ ORⅠ(95%CI) | 1.00 | 0.89 (0.64,1.25) | 0.89 (0.63,1.26) | 0.84 (0.60,1.19) | 0.349 |
| ^c^ ORⅡ(95%CI) | 1.00 | 1.09 (0.71,1.68) | 1.06 (0.69,1.64) | 1.00 (0.65,1.53) | 0.352 |

^a^: Median intake in male/female controls at each quantile after energy correction.

^b^. No factor adjusted.

^c^. Adjusted for age, sex, marital status, educational level, occupation, smoking, alcohol consumption, tea-drinking, economic conditions, energy intake, physical activity, and family history of T2DM.

**Supplementary Table S2.** Odds ratios (95%CIs) of T2DM for quartiles of fresh beans and mix beans in Guangzhou, China

|  | Quartiles of dietary energy-adjusted intake | | | |  |
| --- | --- | --- | --- | --- | --- |
| Food group | Q1(Referent) | Q2 | Q3 | Q4 (highest) | p - trend |
| Fresh beans |  |  |  |  |  |
| ^a^ Intake (M/F), g/d | 2.4/3.1 | 9.6/11.8 | 19.4/20.9 | 43.4/59.8 |  |
| N (case/control) | 134/192 | 94/192 | 83/192 | 73/192 |  |
| ^b^ ORⅠ(95%CI) | 1.00 | *0.70 (0.50,0.96)* | *0.61 (0.43,0.86)* | *0.53 (0.37,0.76)* | *<0.001* |
| ^c^ ORⅡ(95%CI) | 1.00 | 0.85 (0.57,1.27) | *0.60 (0.39,0.92)* | *0.57 (0.37,0.87)* | *0.006* |
| Mixed beans |  |  |  |  |  |
| ^a^Intake (M/F), g/d | 0.01/0.1 | 0.3/0.7 | 1.0/1.9 | 3.2/5.0 |  |
| N (case/control) | 146/192 | 76/192 | 95/192 | 64/192 |  |
| ^b^ ORⅠ(95%CI) | 1.00 | *0.52 (0.37,0.74)* | *0.62 (0.44,0.87)* | *0.41 (0.28,0.60)* | *<0.001* |
| ^c^ ORⅡ(95%CI) | 1.00 | *0.47 (0.30,0.74)* | *0.56 (0.37,0.87)* | *0.41 (0.26,0.64)* | *<0.001* |

^a^: Median intake in male/female controls at each quantile after energy correction.

^b^. No factor adjusted.

^c^. Adjusted for age, sex, marital status, educational level, occupation, smoking, alcohol consumption, tea-drinking, economic conditions, energy intake, physical activity, and family history of T2DM.

**Supplementary Table S3.** Odds ratios (95%CIs) of T2DM for quartiles by subgroups of fruits in Guangzhou, China

|  | Quartiles of dietary energy-adjusted intake | | | |  |
| --- | --- | --- | --- | --- | --- |
| Food group | Q1(Referent) | Q2 | Q3 | Q4 (highest) | p - trend |
| Citrus fruits | | | | | |
| ^a^ Intake (M/F), g/d | 2.2/2.3 | 9.0/9.8 | 20.6/22.1 | 79.0/73.2 |  |
| N (case/control) | 127/192 | 89//192 | 67/192 | 101/192 |  |
| ^b^ ORⅠ (95%CI) | 1.00 | 1.35 (0.56,3.30) | 0.61 (0.19,2.01) | 0.78 (0.27,2.28) | 0.514 |
| ^c^ ORⅡ (95%CI) | 1.00 | 1.69 (0.54,5.27) | 0.50 (0.13,1.94) | 0.56 (0.13,2.36) | 0.358 |
| Apples, pears, peaches, pineapples, plums | | | | | |
| ^a^ Intake (M/F), g/d | 5.5/8.0 | 20.4/31.2 | 49.2/65.9 | 133.0/142.6 |  |
| N (case/control) | 146/192 | 103/192 | 77/192 | 58/192 |  |
| ^b^ ORⅠ (95%CI) | 1.00 | 0.42 (0.15,1.16) | 0.51 (0.16,1.63) | 0.39 (0.11,1.31) | 0.121 |
| ^c^ ORⅡ (95%CI) | 1.00 | 0.55 (0.12,2.64) | 0.22 (0.04,1.42) | 0.54 (0.09,3.26) | 0.052 |
| Persimmons, mangoes, durian and melon fruits | | | | | |
| ^a^ Intake (M/F), g/d | 2.5/2.3 | 8.5/7.1 | 19.7/14.5 | 76.9/48.2 |  |
| N (case/control) | 136/192 | 113/192 | 67/192 | 67/192 |  |
| ^b^ ORⅠ (95%CI) | 1.00 | 1.06 (0.46,2.43) | 0.56 (0.14,2.31) | 0.80 (0.27,2.43) | 0.537 |
| ^c^ ORⅡ (95%CI) | 1.00 | 1.27 (0.46,3.53) | 0.64 (0.09,4.81) | 0.81 (0.18,3.65) | 0.705 |
| Grapes, lichi, and longan | | | | | |
| ^a^ Intake (M/F), g/d | 1.6/1.5 | 5.6/5.3 | 11.5/11.1 | 43.7/41.1 |  |
| N (case/control) | 163/192 | 78/192 | 84/192 | 59/192 |  |
| ^b^ ORⅠ (95%CI) | 1.00 | 0.71 (0.26,1.94) | 0.99 (0.32,3.10) | 0.51 (0.13,2.00) | 0.371 |
| ^c^ ORⅡ (95%CI) | 1.00 | 1.02 (0.26,3.94) | 0.53 (0.11,2.50) | 0.26 (0.05,1.29) | 0.093 |
| Bananas | | | | | |
| ^a^ Intake (M/F), g/d | 1.2/1.2 | 6.5/5.6 | 15.2/15.8 | 54.5/60.1 |  |
| N (case/control) | 144/192 | 71/192 | 82/192 | 87/192 |  |
| ^b^ ORⅠ (95%CI) | 1.00 | 0.70 (0.27,1.79) | 0.93 (0.34,2.56) | 1.00 (0.37,2.66) | 0.918 |
| ^c^ ORⅡ (95%CI) | 1.00 | 0.72 (0.23, 2.26) | 0.34 (0.07, 1.57) | 1.13 (0.33,3.88) | 0.865 |
| Other fruits | | | | | |
| ^a^ Intake (M/F), g/d | 0.0/0.3 | 0.6/2.1 | 3.7/6.5 | 25.4/36.1 |  |
| N (case/control) | 109/192 | 78/192 | 88/192 | 109/192 |  |
| ^b^ ORⅠ (95%CI) | 1.00 | 0.71 (0.49,1.02) | 0.81 (0.57,1.14) | 0.99 (0.71,1.38) | 0.855 |
| ^c^ ORⅡ (95%CI) | 1.00 | 0.77 (0.49,1.21) | 0.74 (0.48,1.13) | 0.90 (0.60,1.37) | 0.604 |

^a^: Median intake in male/female controls at each quantile after energy correction.

^b^. No factor adjusted.

^c^. Adjusted for age, sex, marital status, educational level, occupation, smoking, alcohol consumption, tea-drinking, economic conditions, energy intake,physical activity, and family history of T2DM.

**Supplementary Table S4.** Food items included in the 30 food groups for food composition and factor analysis

| No. | Energy adjusted food items | Food items included |
| --- | --- | --- |
| 1 | Grains^*^ | All items of cereals except whole-wheat bread, including rice (including glutinous rice products), porridge, oatmeal, corn flakes, noodles, white bread or steamed bun, white bread or hamburger, barbecued pork buns, dumplings, Chinese cakes such as radish cakes and water chestnut cakes, cakes, fried snacks, biscuits. |
| 2 | Whole grains ^*^ | Whole-wheat bread |
| 3 | Vegetables with dark color | Cabbage, mustard or cabbage mustard and broccoli, Chinese cabbage, leaf lettuce, lettuce and endive, spinach, edible amaranth and water spinach, other dark green leafy vegetables, onions, scallions, garlic, tomatoes, green and red peppers or bell pepper, carrot. |
| 4 | Vegetables with light color | Chinese cabbage, baby cabbage, broccoli, cauliflower, celery, winter melon, zucchini, cucumber, loofah, eggplant, pumpkin, bitter gourd, white radish, green radish. |
| 5 | Root vegetable | Potatoes, sweet potatoes, taro, lotus root, waternut, arrowroot, Chinese yam, yam bean, corn. |
| 6 | Pickled vegetables | Various pickled Chinese cabbages, pickles, preserved vegetables. |
| 7 | Soybeans and related products ^*^ | Hard tofu, fried tofu, soft tofu, dried tofu, smoked bean curd, soy chicken, bean curd shin, beancurd sheet, etc., soy milk, beancurd jelly, fresh soybeans, baked beans, soybeans. |
| 8 | Mixed beans^*^ | Mung beans, red beans, rice beans, lentils. |
| 9 | Fresh beans | Peas, broad beans, etc., beans, green beans, snow beans, honey beans, lentils, sword bean, soybean sprouts, green bean sprouts. |
| 10 | Fruits with dark color | Citrus, mango, persimmon, papaya. |
| 11 | Fruits with light color | Apple, pear, peach, plum, pineapple, banana, concord grape, grape, lychee, longan, cantaloupe, watermelon, etc., durian, other fruits, such as guava, sugar apple, dragon fruit, mangosteen, carambola, bayberry. |
| 12 | Red meat and related products | Semi-fat lean pork, lean pork, beef, lamb, pettitoes, pigskin, pork tripe, beef tripe, heart, tongue, pig intestines, liver, kidney, brain, chicken giblets. |
| 13 | Pickled red meat | preserved meat, sausage, sausage, ham, luncheon meat, ham sausage, liver sausage, etc.. |
| 14 | Poultry meat with skin | Chicken, duck, and goose with skin (including chicken wings, chicken legs), chicken feet. |
| 15 | Poultry meat without skin | Chicken, duck, and goose without skin. |
| 16 | Freshwater fish | Grass carp, black carp, bullhead, crucian, carp, dace, mandarin fish. |
| 17 | Marine fish | Marine fish, such as pomfret and hairtail. |
| 18 | Mollusks and shellfish | Squid, cuttlefish, scallop, dried scallop, shellfish, shrimp, crab. |
| 19 | Pickled fish | Pickled salted fish, small fish with bones, canned fish . |
| 20 | Eggs | Eggs, duck eggs. |
| 21 | High-fat dairy products ^*^ | Whole milk, whole milk powder, milk tea, milkshake, cheese, ice cream, ice-cream cone, yogurt. |
| 22 | Low-fat dairy products ^*^ | Skimmed milk, skimmed milk powder . |
| 23 | Nuts | Peanuts, cashews, walnuts, ginkgo, almonds, pistachios, sesame (sesame paste). |
| 24 | Fungi and seaweed food | Various fresh mushrooms, fungus, and black fungus. |
| 25 | Carbonated drinks | Cola and other soda water. |
| 26 | Fruit juice | Orange juice, apple juice, grape juice, coconut juice, and other juices. |
| 27 | Coffee | Coffee. |
| 28 | Slow-cooked soup | Slow-cooked soup. |
| 29 | Sugar | Sugar consumption in the past year (including white sugar, brown sugar, candy). |
| 30 | Edible oil | Cooking oil consumption per day. |

* the food group was combied by dry weight

**Supplementary Table S5. List of factor loading scores for food items in factor analysis** ^a^

| Pattern 1 |  | Pattern 2 |  | Pattern 3 |  | Pattern 4 |  |
| --- | --- | --- | --- | --- | --- | --- | --- |
| Dietary pattern with high intake of light-colored vegetables and low intake of grains | Factor loading | High fruit dietary pattern | Factor loading | High-meat and low-grain dietary pattern | Factor loading | Dietary pattern with high intake of dark-colored vegetable | Factor loading |
| (36.94)^b^ |  | (13.08) ^b^ |  | (10.37) ^b^ |  | (7.83) ^b^ |  |
| Grains | -0.810 | Grains | -0.105 | Grains | -0.463 | Light-colored vegetables | 0.212 |
| Light-colored vegetables | 0.555 | Light-colored vegetables | -0.145 | Light-colored vegetables | -0.227 | Dark-colored fruits | 0.102 |
| Nuts | 0.403 | Root vegetables | 0.186 | Red meat | 0.909 | Dark-colored vegetables | 0.990 |
| Whole milk | 0.290 | Coffee | 0.102 | Pickled red meat | 0.139 |  |  |
| Root vegetables | 0.263 | Poultry without skin | 0.114 | Mollusks and shellfish | 0.110 |  |  |
| Eggs | 0.212 | Light-colored fruits | 0.928 | Pickled animal food | 0.109 |  |  |
| Coffee | -0.189 | Dark-colored fruits | 0.614 |  |  |  |  |
| Fungi and seaweeds | 0.184 | Total intake of cooking oil | -0.152 |  |  |  |  |
| Mixed beans | 0.165 | Freshwater fish | 0.145 |  |  |  |  |
| Low-fat milk | 0.131 | Red meat | -0.155 |  |  |  |  |
| Poultry without skin | 0.121 | Dark-colored vegetables | 0.102 |  |  |  |  |
| Light-colored fruits | 0.148 |  |  |  |  |  |  |
| Red meat | -0.183 |  |  |  |  |  |  |

^a^ The factor loading of each common factor is obtained by the maximum orthogonal rotation of the variation. To simplify the table, it only lists food or food groups with an absolute value of factor loading ≥0.1.

^b^ The data in brackets represent the contribution rate (%). The cumulative contribution rate of the four dietary patterns is 68.2%.
